# Multiple Sclerosis Lesion Segmentation in Brain MRI Using Inception Modules Embedded in a Convolutional Neural Network

**DOI:** 10.1155/2021/4138137

**Published:** 2021-08-04

**Authors:** Shahab U. Ansari, Kamran Javed, Saeed Mian Qaisar, Rashad Jillani, Usman Haider

**Affiliations:** ^1^Faculty of Computer Science and Engineering, Ghulam Ishaq Khan Institute of Engineering Sciences and Technology, Topi, Pakistan; ^2^National Centre of Artificial Intelligence (NCAI), Saudi Data and Artificial Intelligence Authority (SDAIA), Riyadh, Saudi Arabia; ^3^Electrical and Computer Engineering Department, Effat University, Jeddah 22332, Saudi Arabia; ^4^Communication and Signal Processing Lab, Energy and Technology Research Center, Effat University, Jeddah 22332, Saudi Arabia

## Abstract

Multiple sclerosis (MS) is a chronic and autoimmune disease that forms lesions in the central nervous system. Quantitative analysis of these lesions has proved to be very useful in clinical trials for therapies and assessing disease prognosis. However, the efficacy of these quantitative analyses greatly depends on how accurately the MS lesions have been identified and segmented in brain MRI. This is usually carried out by radiologists who label 3D MR images slice by slice using commonly available segmentation tools. However, such manual practices are time consuming and error prone. To circumvent this problem, several automatic segmentation techniques have been investigated in recent years. In this paper, we propose a new framework for automatic brain lesion segmentation that employs a novel convolutional neural network (CNN) architecture. In order to segment lesions of different sizes, we have to pick a specific filter or size 3 × 3 or 5 × 5. Sometimes, it is hard to decide which filter will work better to get the best results. Google Net has solved this problem by introducing an inception module. An inception module uses 3 × 3, 5 × 5, 1 × 1 and max pooling filters in parallel fashion. Results show that incorporating inception modules in a CNN has improved the performance of the network in the segmentation of MS lesions. We compared the results of the proposed CNN architecture for two loss functions: binary cross entropy (BCE) and structural similarity index measure (SSIM) using the publicly available ISBI-2015 challenge dataset. A score of 93.81 which is higher than the human rater with BCE loss function is achieved.

## 1. Introduction

**Figure 1:**
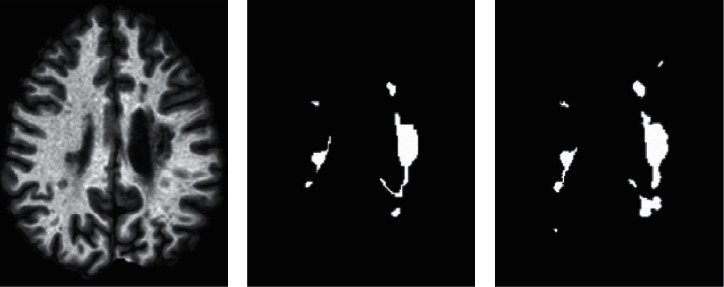
Manual segmentation of MS lesions: (a) T1w MRI, (b) manual segmentation by rater 1, and (c) manual segmentation by rater 2.

Multiple sclerosis (MS) is a chronic disease that damages the nerves in the spinal cord, brain, and optic nerves. Axons in the brain are covered with a myelin sheath. Demyelination is a process in which the myelin sheaths start falling off and develops lesions in brain nerves. Millions of people are affected by MS worldwide which is mainly found in young people between 20 and 50 years of age. The symptoms caused by this disease are fatigue, memory problem, the problem in concentration, weakness, loss of balance, loss of vision, and many others. Diagnosing and treating this disease is very challenging because of its variability in its clinical expression. These lesions can be traced in magnetic resonance imaging (MRI) using different sequences. Many features such as a volume and location are very important biomarkers for tracking the progression of the disease. Manually segmenting these lesions by expert radiologists is the most common practice in clinics, but this is tiresome, time consuming, and error prone.  Figure 1 shows the manual segmentation of MS lesions by two raters in one slice of a brain MRI.

In recent years, automatic segmentation of MS lesions using convolutional neural networks (CNNs) have been investigated [1–5]. CNNs learn subtle features from the raw image data to facilitate 2D pixel (or 3D voxel) classification that ultimately leads to image segmentation. However, there is no one-fit-for-all CNN model that could work for every classification problem or data. An expert knowledge has to be incorporated during the design phase of the CNN model based on the nature of the application and the data. Complex problems such as MS lesion segmentation require careful selection of the CNN architecture and training model for an optimum solution. In addition, automatic segmentation of an MS lesion in MRI may be challenging due to the following:

- The lesion size and location are highly variable
- The edges between anatomical objects are not well defined in MR images due to low contrast
- The MR image of clinical quality may have imaging artifacts such as noise and inhomogeneity

In this work, we are proposing a novel CNN architecture for MS lesion segmentation. The MS lesions vary tremendously in size and shape, and sometime, it is difficult to detect in brain MR images. To address this particular challenge, inception modules, originally introduced by Google in GoogLeNet, are added in the CNN model [6]. The significance of the inception module lies in using multiple kernels of different sizes in parallel in an efficient way. This smart approach captures features of varying magnitude in the input data without overburdening the network with additional computations. The proposed model is trained for two loss functions, binary cross entropy (BCE) and structural similarity index measure (SSIM). The BCE loss function tries to maximize the difference of the probability distribution between two classes, in this case, lesion and nonlesion voxels [7]. SSIM, on the other hand, is a perception-based loss function that quantifies the similarity between two images [8].

The proposed solution for the MS lesion segmentation in brain MRI offers the following attributes:

- Introduction of inception modules embedded in the CNN architecture for the segmentation of MS lesions with different shapes and sizes
- Comparison of MS lesion segmentation results using BCE and SSIM loss functions
- Improvement of performance of the proposed architecture in terms of the Dice coefficient, positive predicted value, lesion-wise true positive rate, and volume difference of the segmented lesions compared to the gold standard

### 1.1. Literature Review

In past decade, deep neural networks have shown promising results in the segmentation of MS lesions in brain MR images. In [9], a novel architecture for segmenting MS lesions in magnetic resonance images by using a deep 3D convolutional encoder with the connections of shortcut in pathways was proposed. The method was evaluated on publicly available data from ISBI-2015 [10] and MICCAI-2008 [11] challenges. Authors compared their method with other five available approaches used for MS lesion segmentation. The final results show that their method outperformed the previous existing methods for MS lesion segmentation. In [12], the authors used a fully automatic multiview CNN approach for segmenting a multiple sclerosis lesion in longitudinal MRI data and tested on the ISBI-2015 dataset. Various deep learning techniques for the medical image analysis are presented in [13].

Valverde et al. have proposed a novel architecture for segmentation of a white matter (WM) lesion in multiple sclerosis (MS) using small number of imaging data [14]. This approach proposed a cascaded CNN model working on 3D MRI patches from FLAIR and T1w modalities. In this method, the output of the first network is retrained on the second network in series to reduce misclassification from the first network. The proposed model score is evaluated on the publicly available dataset of MICCAI-2008 and outperformed all the participant approaches. Roy et al. proposed a fully convolutional neural network (FCNN) to segment WM lesions in multicontrast MR images using multiple convolutional pathways [15]. The first pathway of the CNN contains dual convolutional filters for two image modalities. In the second pathway, the convolutional filters are applied to the output of the first pathway which are in parallel and concatenated. This method was evaluated on the ISBI-2015 dataset. A novel approach of using a fully 2D CNN to segment MS lesions in MR images is proposed in [16]. Maleki et al. have investigated the use of a CNN model for the detection and segmentation of MS lesions [17].

In recent studies, a multimodal MRI dataset in tissue segmentation has shown promising results. In a recent work for brain tumor segmentation, a deep multitask learning framework that performs a performance test on multiple BraTS datasets was shown [18]. The authors claimed improvement over the traditional V-Net framework by using a structure of two parallel decoder branches. The original decoder performs segmentation, and the newly added decoder performs the auxiliary task of distance estimation to make more accurate segmentation boundary. A total loss function is introduced to combine the two tasks with a gamma factor to reduce the focus on the background area and set different weights for each type of label to alleviate the problem of category imbalance. Zhang et al. proposed the ME-Net model and obtained promising results using the BraTS 2020 dataset [19]. Four encoder structures for the four modal images of brain tumor MRI were employed with skip-connections. The combined feature map was given as input to the decoder. The authors also introduced a new loss function, that is, Categorical Dice, and set different weights for different masks. In another study, a 3D supervoxel-based learning method was proposed that demonstrated promising results in the segmentation of brain tumor [20]. The added features from multimodal MRI images greatly increased the segmentation accuracy. In another earlier study, Gabor texton feature, fractal analysis, curvature, and statistical intensity features from superpixels were used to segment tumors in multimodal brain MR images using extremely randomized trees (ERTs) [21]. The experimental results demonstrated the high detection and segmentation performance of the proposed method. Soltaninejad et al. proposed a method that used machine-learned features learned by fully convolutional networks (FCNs) and texton-based histograms as hand-crafted features [22]. The random forest (RF) classifier was then employed for the automated segmentation of brain tumor in the BraTS 2017 dataset.

Segmentation results can be greatly affected by the quality of the MRI images. Low resolution, intensity variations, and image acquisition noise hamper the accuracy of a segmentation task. Jin et al. proposed a deep framework for the segmentation of prostate cancer [23]. They had shown that the segmentation results were greatly improved by using bicubic interpolation and improved version of 3D V-Net. The bicubic interpolation of the input data helped in enhancing the relevant features required for prostate segmentation. Recently, attention-based methods have gained reputation in the segmentation of small but discrete objects in MRI images. In a study, for the enhancement of left atrium scars, a dilated attention network was used [24]. The proposed approach improved the accuracy of the scar segmentation to 87%. Liu et al. proposed a spatial attentive Bayesian deep learning network for the automatic segmentation of the peripheral zone and transition zone of the prostate with uncertainty estimation [25]. This method outperformed the state-of-the-art methods.

The heterogeneity of MS lesions poses a challenge for the detection and segmentation in MR images. An attention-based fully CNN has also been used in the segmentation of prostate zones [26]. The authors in this work have proposed a novel feature pyramid attention mechanism to cope with heterogeneous prostate anatomy. Raschke et al. developed a statistical method to analyze heterogeneity of brain tumors in multimodal MRI [27]. The approach presented in the paper does not make any assumption on the probability distribution of the MRI data and prior knowledge of the location of tumors. This, according to the authors, gives an advantage for tumor segmentation of varying sizes and spatial locations. The proposed method consist of two deep subnetworks in which the first one was an encoding network that was responsible of extracting feature maps and the second was a decoding network and was responsible for upsampling feature maps. The proposed FCNN was evaluated on an ISBI-2015 dataset.

## 2. Proposed Methodology

As mentioned earlier, the shape and size of MS lesions vary dramatically. To detect these lesions using machine learning techniques is a challenging task. In the proposed methodology, a CNN model with inception modules is employed to automatically segment MS lesions in brain MRI. Filters of multiple sizes used in the inception modules capture features of MS lesions of different sizes. Prior to CNN model training, the images in the dataset are first preprocessed to remove image noise, intensity inhomogeneity, variability of intensity ranges, and the presence of nonbrain tissues. In this work, preprocessed ISBI-2015 image data have been used.

### 2.1. Dataset

**Figure 2:**
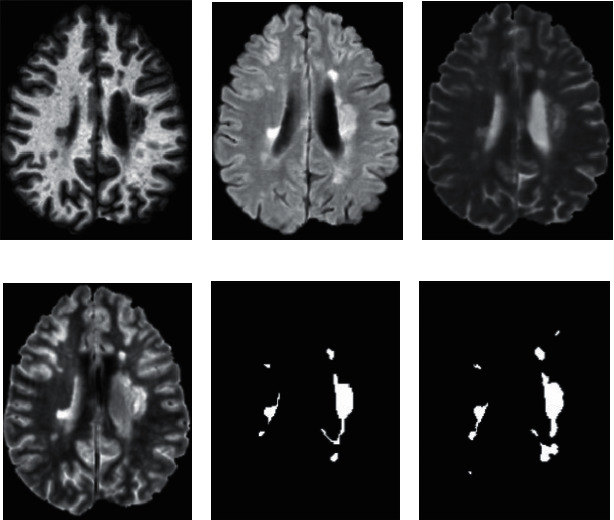
Sample of the ISBI dataset: (a) T1w, (b) FLAIR, (c) T2w, and (d) PDw, (e) manual delineation by rater 1, and (f) manual delineation by rater 2.

The proposed algorithm uses the dataset of ISBI-2015 challenge [10] which is grouped in two categories, training and testing data. The training data are named ISBI-21 and are available publicly with 21 MRI images from 5 patients. In the training set, MR brain images of four patients with 4 time points and one with 5 time points with a gap of approximately a year are gathered. The test data are named as ISBI-61 which are not available publicly and have 14 subjects with 61 images. Each subject in the testing set has 4-5 time points, and each time point has a gap of approximately a year. These images contain longitudinal scans of all five patients, as shown in Figure 2. During training, we used 80 percent of the total patches of 100 × 100 size for training and the remaining 20 percent for validation.

### 2.2. Proposed Deep Network Architecture

In the CNN architecture, a kernel size and type of filters have to be selected carefully so that it can learn all the features which are useful in the classification of objects. Generally, filters of different sizes and pooling schemes are employed in different CNN layers in order to learn most present features in the data. The inception module, however, uses multiple kernels in each layer in parallel and then pools the features [28]. In the proposed framework, we have investigated the efficacy of inception modules embedded in the CNN model for the segmentation of MS lesions.

#### 2.2.1. CNN Model

**Figure 3:**
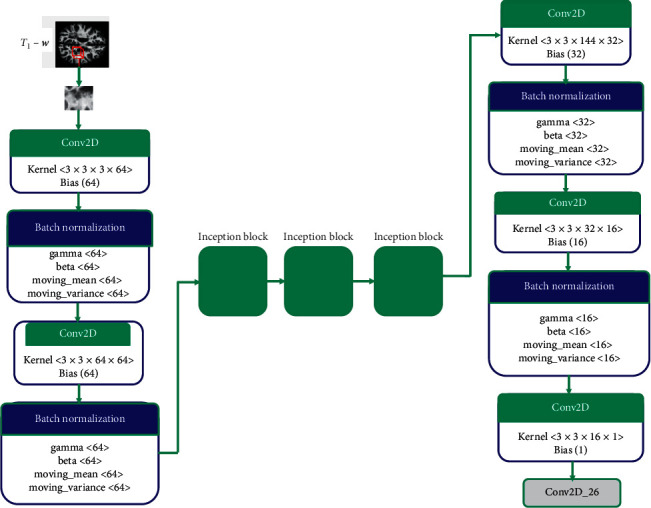
Proposed deep network architecture for MS lesion segmentation in brain MRI.

In the proposed method for segmentation of multiple sclerosis disease, we incorporated three inception modules in our CNN model. Each module consists of 1 × 1, 3 × 3, 5 × 5, max pooling, and average pooling. The CNN model consists of two convolution layers with 64 feature maps followed by inception modules and then three convolutions layers. The final layer has one feature map for the prediction of lesion and nonlesion voxels.  Figure 3 shows the complete architecture with inception modules embedded in the CNN layers. The model is trained with two different loss functions, i.e., binary cross entropy (BCE) and structural similarity index measure (SSIM). BCE is a measure of the difference between two probability distributions for a given random variable or a set of events and is used in binary classification tasks, whereas SSIM is a perceptual metric that quantifies image quality degradation caused by losses in data compression. For high similarity in images, the value of BCE is low and the value of SSIM is high.

#### 2.2.2. Inception Module

**Figure 4:**
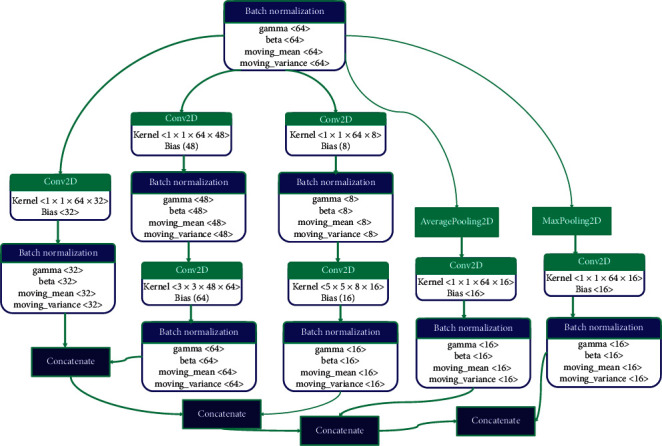
Inception block in the proposed CNN architecture.

The fundamental idea behind the GoogLeNet is the introduction of inception modules or inception blocks in the CNN architecture. In CNN, the feature maps learned from the previous layer are given as input to the next layer. The inception module takes the previous layer output and passes it to four different filter operations in parallel, as shown in Figure 4. The feature maps from all the filters are then concatenated to form the final output. The fundamental idea of using a 1 × 1 kernel in the inception module is just to shrink the depth of the feature maps [29]. The 1 × 1 convolutions preserve the parameters spatially that can be used when needed. This strategy in the inception module can lower the dimensions of the feature maps which can eventually drop the computational cost.

### 2.3. Loss Functions

The proposed model is trained for two loss functions, binary cross entropy (BCE) and structural similarity index measure (SSIM). The BCE loss function tries to maximize the difference of the probability distribution between two classes, in this case, lesion and nonlesion voxels. It measures the performance of a classification model whose output is the probability between 0 and 1, i.e., the output of sigmoid activation. Mathematically, BCE loss for an output *y* with probability *p* can be computed as

(1) $$\begin{array}{c} \text{BCE}=-y\;\log\;p-\left(1-y\right)\log\left(1-p\right). \end{array}$$

SSIM is a perception-based loss function that quantifies the similarity between two images. In SSIM, similarity between two images can be computed using a statistical model. Let *μ*_*x*_ and *μ*_*y*_ be the means, *σ*_*x*_ and *σ*_*y*_ be the variances, and *σ*_*xy*_ be the covariance of the two images *x* and *y*; then,

(2) $$\begin{array}{c} \text{SSIM}\left(x,y\right)=\frac{\left(2\mu_{x}\mu_{y}+C_{1}\right)+\left(2\sigma_{xy}+C_{2}\right)}{\left(\mu_{x}^{2}+\mu_{y}^{2}+C_{1}\right)\left(\sigma_{x}^{2}+\sigma_{y}^{2}+C_{2}\right)}, \end{array}$$

where *C*_1_ and *C*_2_ are regularization constants.

### 2.4. Model Implementation

The CNN model is implemented in Python using Keras [30] with TensorFlow library [31]. All the experiments were performed on the Nvidia GeForce RTX 2080 GPU. The deep network is trained end to end using patches. During the training phase of the CNN model, the patches are extracted from each slice in MR images. The training set is divided into two subsets, one for training the network and the other for validating the results. The optimization technique employed to update the parameters in the model is the Adam method [32]. In neural network parameter optimization, the Adam method shows better convergence. The hyperparameters used during network training include the fixed learning rate of 0.0001 for 50 epochs. These parameters' setting has produced sufficient convergence to optimal network parameters without overfitting the data. The size of the minibatch is set to 64, and each minibatch includes random number of patches. The best model from the validation set is selected at the 24^th^ epoch which takes 48 hours on the GPUs.

## 3. Results and Discussion

### 3.1. Performance Metrics

Standard performance metrics for the assessment of the proposed CNN model have been employed. The Dice similarity coefficient measures reproducibility of segmentation as a statistical validation of manual annotation. Another similar metric is the Jaccard similarity index that gives the intersection between the machine segmentation and the ground truth. Positive predicted value is the probability that people with a positive screening test result indeed have the condition of interest. The portion of positive voxels in ground truth that is also identified as positive in the automatic segmentation is captured by true positive rate. Lesion-wise true/false positive rate is the number of lesions that overlap/do not overlap in automatic segmentation and the ground truth. The difference is volume of automatic segmentation, and the ground is another important metric for the assessment of the performance of the CNN model. The Pearson correlation coefficient computes the correlation between the automatic segmentation and the ground truth. The overall score gives the average of the combined effect of all these performance metrics in a single number.  Table 1 shows formulas for these performance metrics.

### 3.2. Feature Learning by Inception Modules

As suggested by the literature, the proposed CNN model is trained on T1w, T2w, and FLAIR sequences of the MRI data.  Table 2 shows quantitative results for automatic MS lesion segmentation in MRI using the BCE loss function for test images at time points *TP*. Although, in the results, both Dice and Jaccard similarity indices are reported, they both convey the same information. The performance metrics observed for the proposed CNN model have significantly outperformed when compared with the existing techniques, as shown in Table 3. Kernels of different sizes used in the inception modules help in extracting discriminative features for the automatic segmentation of MS lesions and background tissues in brain MRI. The most present features are ultimately pooled using max pooling and average pooling at various stages of the inception modules. The number of inception modules used in the CNN model is also very crucial in the architecture design. Using too many inception modules in MS lesion segmentation has degraded the results due to overfitting the model to the data. Also, poor results are obtained when the number of inception modules has been lowered. This may correspond to underfitting the CNN model for the segmentation of MS lesions. Experiments have also confirmed that a mix of average pooling and max pooling works better by keeping the most present features in the high-level feature maps and averaging them in the low-level feature maps. The authors suggest that, for a specific application, the number and placement of inception modules, filter size, and pooling strategy have to be selected accordingly.

### 3.3. Comparison of BCE and SSIM Loss Functions

**Figure 5:**
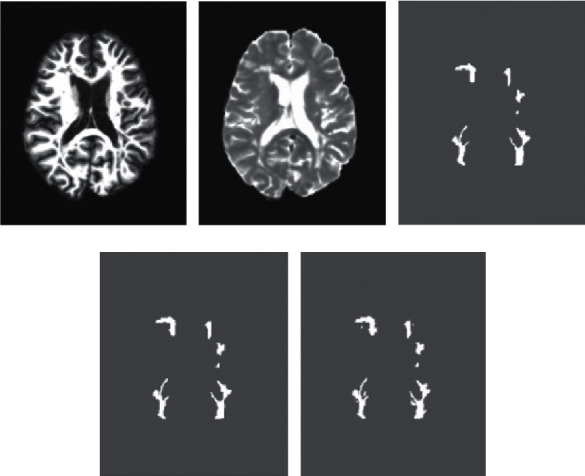
Comparison of the segmentation results when using BCE and SSIM loss functions. (a) T1w. (b) T2w. (c) Rater 1. (d) BCE. (e) SSIM.

Two loss functions in training the proposed CNN model have been used, BCE and SSIM. Tables 2 and 4 report the quantitative results for the two loss functions.  Table 5 gives the comparison of the two loss functions on the basis of the average values of the results. In the MS lesion classification, the BCE loss function seems to work better than SSIM. This sounds very intuitive as BCE tries to evaluate the difference in the maximum likelihood between the predictions and ground truths. SSIM, on the other hand, quantifies the perceptual differences between the predictions and the ground truths. It uses luminance, contrast, and structure features to compute the similarity between two images. The reason why the BCE loss function works better than SSIM is because loss functions also depend on the activation functions used in the output layer. For sigmoid activation, the literature suggests that the BCE loss function is the natural choice due to its accuracy and efficiency. The automatic MS lesion segmentation using BCE and SSIM loss functions is illustrated in Figure 5.

### 3.4. Comparison with Existing Techniques

The proposed methodology is compared with different published techniques for MS lesion segmentation using the ISBI-2015 dataset. The comparison of the results is shown in Table 3. The Dice coefficient, PPV, LTPR, and VD obtained in the proposed methodology show that the model is generalized well for successfully handling new data. The performance of Birenbaum and Greenspan's model, multiview CNN, includes a score of 90.07, DSC of 62.71%, PPV of 78.89%, LTPR of 56.78%, LFPR of 49.75%, and VD of 35.22%. This model produced the best LFPR result among the five techniques compared here. The performance of Litjens' CNN model used was the worst compared to the other techniques. The performance value of score was 86.92, the DSC was 50.09%, PPV was 54.91%, LTPR was 42.88%, LFPR was 57.95%, and VD was 57.07%. The second best performance was shown by the cascaded CNN architecture proposed by Velverde et al. It includes a score of 91.33, DSC of 62.94%, PPV of 78.66%, LTPR of 36.69% LFPR of 15.29%, and VD of 33.84%. The results for the multibranch CNN model proposed by Aslani et al.'s model includes a score of 89.85, DSC of 48.56%, PPV of 74.01%, LTPR of 30.34%, LFPR of 17.08%, and VD of 47.68%. Finally, the performance of the proposed model was the best with a score of 93.81, DSC of 67.11%, PPV of 86.58%, LTPR of 50.60%, LFPR of 12.34%, and VD of 33.35%. The value of LTPR was the only metric that was worse than Valverde's and Aslani's models. The shortcoming in LFPR can further be investigated in the future model of the present work.

## 4. Limitations in Real Clinical Studies

The proposed work is an attempt to prove the efficacy of AI-based techniques in medical applications. In recent years, AI has gained reputation in automating tedious routine works in clinical settings. However, the diversity and inadequacy of the patient data for training a deep network have hampered practical use of AI-based techniques in clinics. As more and more data will become available and as deep neural networks will become more efficient, the practicability of these techniques will definitely improve.

## 5. Conclusions and Future Works

In this work, a CNN model with inception modules is investigated in automatic segmentation of MS lesions in MRI. The CNN model with inception modules seems to pick MS lesions of different sizes and shapes more successfully. The key advantage of inception modules is the use of different kernels such as 1 × 1, 3 × 3, and 5 × 5 that tend to extract salient features in the input of varying sizes. This improves the Dice coefficient, PPV, LTPR, and VD of the segmentation when compared to the existing techniques. These results have outperformed all the existing techniques. The success of Velverde's model can also be attributed to accurate learning of MS lesion features of various sizes and shapes. The performance of Birenbaum and Greenspan's model, multiview CNN, includes a score of 90.07, DSC of 62.71%, PPV of 78.89%, LTPR of 56.78%, LFPR of 49.75%, and VD of 35.22%. This model produced the best LFPR result among the five techniques compared here. The performance of Litjens' CNN model was the worst compared to the other techniques. The performance of the model used had a score of 86.92, DSC of 50.09%, PPV of 54.91%, LTPR of 42.88%, LFPR of 57.95%, and VD of 57.07%. The second best performance was shown by the cascaded CNN architecture proposed by Velverde et al. It includes a score of 91.33, DSC of 62.94%, PPV of 78.66%, LTPR of 36.69%, LFPR of 15.29%, and VD of 33.84%. The results for the model proposed by Aslani et al.'s model, multibranch CNN, includes a score of 89.85, DSC of 48.56%, PPV of 74.01%, LTPR of 30.34%, LFPR of 17.08%, and VD of 47.68%. Finally, the performance of the proposed model was the best with a score of 93.81, DSC of 67.11%, PPV of 86.58%, LTPR of 50.60%, LFPR of 12.34%, and VD of 33.35%. The value of LTPR was the only metric that was worse than Valverde's and Aslani's models. In the present study, we have also discovered that the BCE loss function works better than the SSIM loss function. The intuition behind this behavior of the model is that BCE tries to maximize the differences between the probability distributions predictions and ground truths. SSIM, on the other hand, seems to converge to local minima while quantifying the error loss. Another important reason is the sigmoid activation function used in the output layer for the binary classification. The authors believe this naturally supports the BCE loss function to produce more accurate and efficient results. In the future, this work can be further extended to integrate in different architectures such as the residual network (ResNet), UNet, parallel CNN, and cascaded CNN on multiple datasets which are publicly available. The incorporation of event-driven processing can improve the performance of the suggested solution in terms of computational efficiency and compression [33–36]. Investigation based on this axis is another prospect.

## Figures and Tables

**Table 1:** Performance metrics used in the proposed solution.

Metric	Formula
Dice similarity coefficient	DSC=2TP/(FN+FP+2TP)
Jaccard similarity coefficient	JSC=TP/(TP+FP+FN)
Positive predicted value	PPV=TP/(TP+FP)
True positive rate	TPR=TP/(TP+FN)
Lesion-wise true positive rate	LTPR=LTP/RL
Lesion-wise false positive rate	LFPR=LFP/PL
Volume difference	VD=TP_*s*_ − TP_*gt*_/TP_*gt*_
Pearson correlation coefficient	Cor=cov(*X*, *Y*)/*σ*_*X*_*σ*_*Y*_
Overall score	SC=(1/|*R*|+|*S*|)∑_*R*,*S*_((DSC/8)+(PPV/8)+(1 − LFPR/4)+(LTPR/4)+(Cor/4))

**Table 2:** Quantification of MS lesion segmentation with the BCE loss function.

Subject	TP	Dice	Jaccard	PPV	TPR	LFPR	LTPR	VD
test01	1	0.6639	0.4969	0.8991	0.5263	0.1356	0.5068	0.4147
test01	2	0.6916	0.5286	0.9131	0.5566	0.0806	0.5128	0.3904
test01	3	0.682	0.5174	0.8845	0.5549	0.1452	0.5	0.3726
test01	4	0.6732	0.5074	0.9226	0.5299	0.1034	0.4667	0.4256
test02	1	0.6933	0.5306	0.7548	0.6411	0.1176	0.4653	0.1507
test02	2	0.6823	0.5178	0.8229	0.5828	0.087	0.4969	0.2918
test02	3	0.664	0.497	0.8241	0.5559	0.0638	0.4867	0.3254
test02	4	0.6409	0.4716	0.8529	0.5134	0.0631	0.5411	0.3981
test02	5	0.7099	0.5502	0.8444	0.6123	0.1277	0.4157	0.2748
test03	1	0.4949	0.3288	0.8944	0.3421	0.125	0.3056	0.6175
test03	2	0.5132	0.3451	0.9271	0.3548	0.1379	0.3333	0.6173
test03	3	0.4988	0.3322	0.9457	0.3387	0	0.4375	0.6419
test03	4	0.5838	0.4122	0.9242	0.4266	0.08	0.4667	0.5384
test04	1	0.8168	0.6903	0.8693	0.7702	0.1154	0.6944	0.114
test04	2	0.7928	0.6567	0.8205	0.7668	0.36	0.5172	0.0654
test04	3	0.8067	0.676	0.8099	0.8035	0.08	0.7586	0.0078
test04	4	0.7999	0.6665	0.8095	0.7905	0.2759	0.697	0.0234
Average		0.6711	0.5133	0.8658	0.5686	0.1234	0.5060	0.3335

**Table 3:** Comparison with the existing techniques.

Method	SC	DSC	PPV	LTPR	LFPR	VD
Birenbaum and Greenspan [12]	90.07	0.6271	0.7889	0.5678	0.4975	0.3522
Litjens et al. [13]	86.92	0.5009	0.5491	0.4288	0.5765	0.5707
Valverde et al. [14]	91.33	0.6294	0.7866	0.3669	0.1529	0.3384
Aslani et al. [16]	89.85	0.4856	0.7402	0.3034	0.1708	0.4768
Proposed	90.84	0.6306	0.7888	0.5736	0.2512	0.3444

**Table 4:** Quantification of MS lesion segmentation with the SSIM loss function.

Subject	TP	Dice	Jaccard	PPV	TPR	LFPR	LTPR	VD
test01	1	0.6061	0.4348	0.866	0.4662	0.0408	0.4247	0.4617
test01	2	0.6296	0.4595	0.8677	0.4941	0.0702	0.4487	0.4306
test01	3	0.6179	0.447	0.85	0.4853	0.0556	0.4024	0.429
test01	4	0.6194	0.4486	0.8814	0.4775	0.0909	0.4267	0.4582
test02	1	0.6608	0.4934	0.7923	0.5667	0.0864	0.375	0.2847
test02	2	0.631	0.4609	0.8246	0.5111	0.101	0.4025	0.3802
test02	3	0.5987	0.4273	0.8224	0.4707	0.0667	0.34	0.4277
test02	4	0.5909	0.4194	0.852	0.4523	0.0467	0.4452	0.4692
test02	5	0.6617	0.4944	0.8427	0.5446	0.0978	0.3614	0.3537
test03	1	0.4394	0.2815	0.7986	0.3031	0.0769	0.3333	0.6205
test03	2	0.4663	0.3041	0.8313	0.3241	0.08	0.4	0.6102
test03	3	0.4597	0.2985	0.852	0.3148	0.0909	0.375	0.6305
test03	4	0.5254	0.3563	0.8287	0.3847	0.2	0.3333	0.5358
test04	1	0.776	0.634	0.8535	0.7115	0.1304	0.5833	0.1664
test04	2	0.762	0.6156	0.8345	0.7012	0.2353	0.4138	0.1597
test04	3	0.7729	0.6299	0.8089	0.74	0.2273	0.5862	0.0852
test04	4	0.7792	0.6383	0.8187	0.7433	0.1667	0.6364	0.0921
test05	1	0.433	0.2763	0.3939	0.4806	0.45	0.2778	0.2201
test05	2	0.4622	0.3006	0.5652	0.391	0.1852	0.4082	0.3082
test05	3	0.5353	0.3655	0.6448	0.4576	0.1951	0.4923	0.2903
test05	4	0.5169	0.3485	0.6145	0.446	0.1923	0.375	0.2742
Average		0.5974	0.4350	0.7830	0.4984	0.1374	0.4210	0.3661

**Table 5:** Quantitative comparison of BCE and SSIM loss functions.

Loss function	SC	DSC	PPV	LTPR	LFPR	VD
BCE	90.84	0.6306	0.7888	0.5736	0.2512	0.3444
SSIM	89.01	0.5934	0.7288	0.4476	0.1935	0.3999

## Data Availability

The dataset, studied in this paper, is publically available at https://biomedicalimaging.org/2015/program/isbi-challenges.

## References

[1] Carass A., Roy S., Jog A. (2017). Longitudinal multiple sclerosis lesion segmentation: resource and challenge. *NeuroImage*.

[2] Egger C., Opfer R., Wang C. (2017). MRI FLAIR lesion segmentation in multiple sclerosis: does automated segmentation hold up with manual annotation?. *NeuroImage: Clinica*.

[3] Sati P., Oh J., Oh J. (2016). The central vein sign and its clinical evaluation for the diagnosis of multiple sclerosis: a consensus statement from the North American imaging in multi ple sclerosis cooperative. *Nature Reviews Neurology*.

[4] García-Lorenzo D., Francis S., Narayanan S., Arnold D. L., Collins D. L. (2013). Review of automatic segmentation methods of multiple sclerosis white matter lesions on conventional magnetic resonance imaging. *Medical Image Analysis*.

[5] Kalincik T., Vaneckova M., Tyblova M. (2012). Volumetric MRI markers and predictors of disease activity in early multiple sclerosis: a longitudinal cohort study. *PLoS One*.

[6] Szegedy C., Liu W., Jia Y. Going deeper with convolutions.

[7] Javed K., Ud Din N., Bae S., Yi J. (2019). Image unmosaicing without location information using stacked GAN. *IET Computer Vision*.

[8] Javed K., Din N. U., Bae S., Maharajan R. S., Seo D., Yi J. UMGAN: generative adversarial network for image unmosaicing using perceptual loss.

[9] Brosch T., Tang L. Y. W., Yoo Y., Li D. K. B., Traboulsee A., Tam R. (2016). Deep 3d convolutional encoder networks with shortcuts for multiscale feature integration applied to multiple sclerosis lesion segmentation. *IEEE Transactions on Medical Imaging*.

[10] Isbi 2015 (2015). International symposium on biomedical imaging. *IEEE Pulse*.

[11] Miccai 2008 Medical image computing and computer-assisted intervention.

[12] Birenbaum A., Greenspan H. (2016). Longitudinal multiple sclerosis lesion segmentation using multi-view convolutional neural networks. *Deep Learning and Data Labeling for Medical Applications*.

[13] Litjens G., Kooi T., Bejnordi B. E. (2017). A survey on deep learning in medical image analysis. *Medical Image Analysis*.

[14] Valverde S., Cabezas M., Roura E. (2017). Improving automated multiple sclerosis lesion segmentation with a cascaded 3d convolutional neural network approach. *NeuroImage*.

[15] Roy S., Butman J. A., Reich D. S., Calabresi P. A., Pham D. L. (2018). Multiple sclerosis lesion segmentation from brain MRI via fully convolutional neural networks. https://arxiv.org/abs/1803.09172.

[16] Aslani S., Dayan M., Storelli L. (2019). Multi-branch convolutional neural network for multiple sclerosis lesion segmentation. *NeuroImage*.

[17] Maleki M., Teshnehlab M., Nabavi M. (2012). Diagnosis of multiple sclerosis (MS) using convolutional neural network (CNN) from MRIs. *Global Journal of Medicinal Plant Research*.

[18] Huang H., Yang G., Zhang W. (2021). A deep multi-task learning framework for brain tumor segmentation. *Frontiers in Oncology*.

[19] Zhang W., Yang G., Huang H. (2021). ME-Net: multi-encoder net framework for brain tumor segmentation. *International Journal of Imaging Systems and Technology*.

[20] Soltaninejad M., Yang G., Lambrou T. (2018). Supervised learning based multimodal MRI brain tumour segmentation using texture features from supervoxels. *Computer Methods and Programs in Biomedicine*.

[21] Soltaninejad M., Yang G., Lambrou T. (2017). Automated brain tumour detection and segmentation using superpixel-based extremely randomized trees in FLAIR MRI. *International Journal of Computer Assisted Radiology and Surgery*.

[22] Soltaninejad M., Zhang L., Lambrou T., Yang G., Allinson N., Ye X. (2017). *MRI Brain Tumor Segmentation and Patient Survival Prediction Using Random Forests and Fully Convolutional Networks International MICCAI Brainlesion Workshop*.

[23] Jin Y., Yang G., Fang Y. (2021). 3D PBV-Net: an automated prostate MRI data segmentation method. *Computers in Biology and Medicine*.

[24] Yang G., Chen J., Gao Z. (2020). Simultaneous left atrium anatomy and scar segmentations via deep learning in multiview information with attention. *Future Generation Computer Systems*.

[25] Liu Y., Yang G., Hosseiny M. (2020). Exploring uncertainty measures in bayesian deep attentive neural networks for prostate zonal segmentation. *IEEE Access*.

[26] Liu Y., Sung K., Yang G. (2019). Automatic prostate zonal segmentation using fully convolutional network with feature pyramid attention. *IEEE Access*.

[27] Raschke F., Barrick T. R., Jones T. L., Yang G., Ye X., Howe F. A. (2019). Tissue-type mapping of gliomas. *NeuroImage: Clinica*.

[28] LeCun Y., Bengio Y., Hinton G. (2015). Deep learning. *Nature*.

[29] Szegedy C., Vanhoucke V., Ioffe S., Shlens J., Wojna Z. Rethinking the inception architecture for computer vision.

[30] Chollet F. (2015). Keras. https://keras.io/.

[31] Abadi M., Agarwal A., Barham P. (2015). Tensorflow: large-scale machine learning on heterogeneous systems. https://arxiv.org/abs/1603.04467.

[32] Kingma D. P., Ba J. (2014). Adam: a method for stochastic optimization. https://arxiv.org/abs/1412.6980.

[33] Qaisar S. M. (2014). Event driven filtering an intelligent technique for activity and power consumption reduction. *Int. J. Circuits Syst. Signal Process.*.

[34] Mina Qaisar S., Sidiya D., Akbar M., Subasi A. (2018). An event-driven multiple objects surveillance system. *International Journal of Electrical and Computer Engineering Systems*.

[35] Qaisar S. M., Subasi A. (2020). Effective epileptic seizure detection based on the event-driven processing and machine learning for mobile healthcare. *Journal of Ambient Intelligence and Humanized Computing*.

[36] Qaisar S. M. (2021). Signal-piloted processing and machine learning based efficient power quality disturbances recognition. *PloS One*.

